# Gold Nanoparticles with Different Particle Sizes for the Quantitative Determination of Chlorpyrifos Residues in Soil by SERS

**DOI:** 10.3390/ijms20112817

**Published:** 2019-06-10

**Authors:** Yong He, Shupei Xiao, Tao Dong, Pengcheng Nie

**Affiliations:** 1 College of Biosystems Engineering and Food Science, Zhejiang University, Hangzhou 310058, China; yhe@zju.edu.cn (Y.H.); 180312@zju.edu.cn (S.X.); 21613052@zju.edu.cn (T.D.); 2 Key Laboratory of Spectroscopy Sensing, Ministry of Agriculture and Rural Affairs, Hangzhou 310058, China; 3 State Key Laboratory of Modern Optical Instrumentation, Zhejiang University, Hangzhou 310058, China

**Keywords:** chlorpyrifos, pesticide residues in soil, gold nanoparticles, particle size, surface-enhanced Raman spectroscopy, partial least squares regression

## Abstract

Chlorpyrifos (CPF) is widely used in the prevention and control of crop pests and diseases in agriculture. However, the irrational utilization of pesticides not only causes environmental pollution but also threatens human health. Compared with the conventional techniques for the determination of pesticides in soil, surface-enhanced Raman spectroscopy (SERS) has shown great potential in ultrasensitive and chemical analysis. Therefore, this paper reported a simple method for synthesizing gold nanoparticles (AuNPs) with different sizes used as a SERS substrate for the determination of CPF residues in soil for the first time. The results showed that there was a good linear correlation between the SERS characteristic peak intensity of CPF and particle size of the AuNPs with an R^2^ of 0.9973. Moreover, the prepared AuNPs performed great ultrasensitivity, reproducibility and chemical stability, and the limit of detection (LOD) of the CPF was found to be as low as 10 μg/L. Furthermore, the concentrations ranging from 0.01 to 10 mg/L were easily observed by SERS with the prepared AuNPs and the SERS intensity showed a good linear relationship with an R^2^ of 0.985. The determination coefficient (Rp^2^) reached 0.977 for CPF prediction using the partial least squares regression (PLSR) model and the LOD of CPF residues in soil was found to be as low as 0.025 mg/kg. The relative standard deviation (RSD) was less than 3.69% and the recovery ranged from 97.5 to 103.3%. In summary, this simple method for AuNPs fabrication with ultrasensitivity and reproducibility confirms that the SERS is highly promising for the determination of soil pesticide residues.

## 1. Introduction

Chlorpyrifos (CPF) is one of the most widely-used organophosphate pesticides that can effectively control a variety of pests, including fungi, viruses, insects and weeds [1,2]. However, the long-term application and even abuse of pesticides results in large-scale and severe soil pollution, which endangers soil organisms and plant growth, destroys soil biodiversity and threatens human health [3,4,5]. Considering the overdose and toxicity of CPF pesticides in agriculture, it is crucial to conduct the efficient determination of CPF residues in soil and other agricultural products. Generally, the limits of detection (LODs) of pesticides in soil are usually at the trace level (mg/kg) or ultra-trace level (μg/kg). In addition, the current methods for detecting pesticides in soil are mainly based on liquid chromatography–mass spectrometry (LC-MS) [6], gas chromatography–mass spectrometry (GC-MS) [7] and gas chromatography (GC) [8]. Although these methods achieve high sensitivities, they are limited by the complicated sample processing, time-consuming detection, expensive reagents and inconvenient instruments [9]. By contrast, spectroscopy techniques have shown great potential in the rapid and online detection of pesticide residues. Some studies such as [10] and [11] have used near infrared reflectance spectroscopy (NIRS) and ultrasensitive fluorescent sensors to detect organochlorine pesticides, and the LODs of pesticides detected by NIRS were high.

Surface-enhanced Raman spectroscopy (SERS) is an extension of normal Raman spectroscopy that relies on the electronic and chemical interactions between the excitation laser, analyte of interest, and SERS substrate [12]. Due to the presence of intense electromagnetic fields localized at the metal surface where molecules are adsorbed, the single aerosol particles on or near the surface of plasmonic nanostructures will attain enhanced factors up to six orders of magnitude [13]. Furthermore, as a powerful spectroscopic technology, it has attracted great attention based on its ultrasensitive and unmarked chemical analysis [14]. The success of SERS is highly dependent on the interaction between the surface of plasmonic nanostructures and adsorbed molecules [15,16]. In recent decades, metal nanometer materials, such as gold nanoparticles (AuNPs), silver nanoparticles (AgNPs) and copper nanoparticles (CuNPs), have been widely reported as SERS-active substrates [17,18]. With the development of SERS substrates, the investigations of SERS based on AuNPs and AgNPs for CPF determination have been reported in many previous studies. It can be seen from Table 1 that AuNPs have been widely used as SERS substrates to detect CPF with LODs generally at the mg/kg level. Although the mentioned results enrich the synthesis method of SERS substrates, these methods still have some shortcomings. Firstly, due to the size and aggregation of AuNPs affected by the concentration of reactants and other conditions, the relationship between SERS sensitivity and the particle size of AuNPs was not elaborated. Secondly, the nanoparticle synthesis procedures, such as nanoimprint, require precise and expensive instruments. Therefore, in order to realize the application and promotion of the SERS technique in pesticide residue detection, it is very important to develop a simple method for synthesizing highly sensitive, reproducible and inexpensive SERS substrates.

In this study, we described a simple method for synthesizing ultrasensitive and reproducible AuNPs with different particle sizes as SERS substrates. The main characteristics are as follows. Firstly, we investigated the relationship between AuNP particle size and the SERS signal intensity at 529, 560, 610, 674, 1100 and 1270 cm^−1^ of the CPF molecule. Secondly, we applied the SERS technique for the determination of CPF in soil for the first time. Using the prepared AuNP substrate, the SERS signals of CPF were qualitatively and quantitatively analyzed. The LOD was estimated to be as low as 10 μg/L. Thirdly, we established the partial least squares regression (PLSR) model between SERS spectra of CPF in soil and CPF concentration. Overall, it is believed that the prepared AuNP substrate is an excellent substrate for sensitive SERS spectroscopy of a class of chemical molecules and can provide a theoretical basis and technical support for the determination of pesticide residues in soil, which is favorable for soil remediation and environmental protection.

## 2. Results and Discussion

### 2.1. SERS Signal Enhancement Based on Different AuNP Substrates 

**Figure 1:**
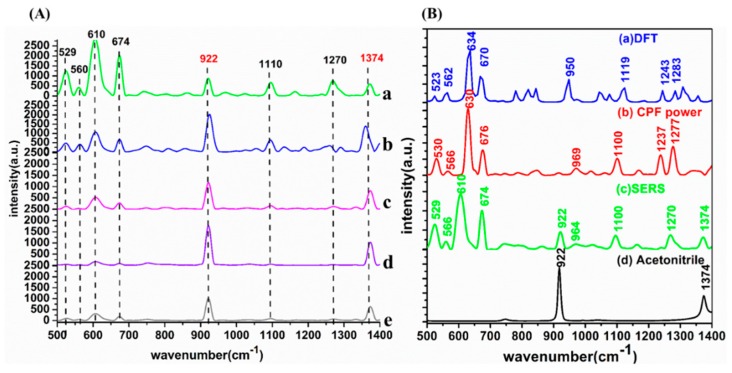
(**A**) The surface-enhanced Raman spectroscopy (SERS) spectra of 10 mg/L chlorpyrifos (CPF) performed with gold nanoparticles (AuNPs) with different amounts of Na_3_C_6_H_5_O_7_: (a) 0.5 mL Na_3_C_6_H_5_O_7_; (b) 1 mL Na_3_C_6_H_5_O_7_; (c) 2 mL Na_3_C_6_H_5_O_7_; (d) 3 mL Na_3_C_6_H_5_O_7_; (e) 4 mL Na_3_C_6_H_5_O_7_. (**B**) The Raman spectral simulation with density functional theory (DFT) calculations: (a) the Raman spectra of CPF powder; (b) the SERS spectra of CPF; (c) the Raman spectra of acetonitrile.

In this paper, a 10 mg/L solution of CPF (C_9_H_11_Cl_3_NO_3_PS) was chosen to estimate the SERS activity and stability of different AuNP substrates. Moreover, for confirming the accuracy of SERS spectra, the Raman spectral simulations were carried out based on density functional theory (DFT) with the assistance of Gaussian v.09 software [27]. The SERS spectra of CPF at 10 mg/L with the different AuNPs is shown in Figure 1A. The Raman spectral simulation with DFT calculations, the Raman spectra of CPF powder, the SERS spectra of CPF and Raman spectra of acetonitrile are shown in Figure 1B. In addition, the vibrational mode of the various peaks of CPF is shown in Table 2. It can be clearly seen in Figure 1B that, except for 922 and 1374 cm^−1^ assigned to acetonitrile, the SERS characteristic peaks were basically consistent with those of CPF powder calculated by DFT, which indicated that the SERS spectra were accurate and reliable.

As shown in Figure 1A, when the amount of Na_3_C_6_H_5_O_7_ increased from 0.5 to 4 mL, the Raman signals decreased sharply and there was only a faint signal when the amount of Na_3_C_6_H_5_O_7_ was 4 mL. In the case of AuNPs with 0.5 mL Na_3_C_6_H_5_O_7_ added, the Raman signal was markedly enhanced and the intensities of the characteristic peaks of the CPF molecule at 529, 610 and 674 cm^−1^ were higher than others. A possible explanation was that the C–Cl and P=S groups performed good affinity for AuNPs, resulting in the higher intensity of the Raman signal. The characteristic peaks at 529 and 610 cm^−1^ were assigned to the P=S and C–Cl stretching vibration, and the characteristic peak at 674 cm^−1^ was assigned to the benzene ring and C–Cl stretching vibration, which were in good agreement with previous studies [26].

### 2.2. Surface Morphology and Optical Absorption of AuNP Substrates

**Figure 2:**
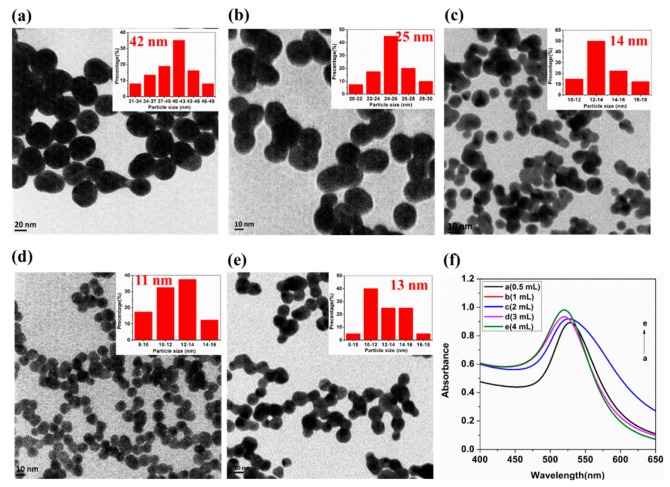
Representative transmission electron microscopy (TEM) images of AuNPs with 0.5 mL Na_3_C_6_H_5_O_7_ (**a**), 1 mL Na_3_C_6_H_5_O_7_ (**b**), 2 mL Na_3_C_6_H_5_O_7_ (**c**), 3 mL Na_3_C_6_H_5_O_7_ (**d**), and 4 mL Na_3_C_6_H_5_O_7_ (**e**), respectively. (**f**) The UV–vis spectrometry of AuNPs.

In order to investigate the relationship between the characteristics and optical absorption properties of AuNPs and SERS signals, the representative transmission electron microscopy (TEM) images (Figure 2a–e) and UV–vis spectrometry (Figure 2f) of AuNPs samples were obtained, respectively. The physical properties of AuNPs are shown in Table 3, where v, α_m_, λ_m_ and r/nm, represent the amount of trisodium citrate, AuNP absorbance at the absorption peak, the AuNPs absorption peak wavelength and AuNP particle size, respectively.

As can be seen, the amount of Na_3_C_6_H_5_O_7_ showed a marked influence on the diameter as well as the optical absorption properties of AuNPs. With the increase of Na_3_C_6_H_5_O_7_ from 0.5 to 4 mL, the average diameter of AuNP particles decreased from 40 to 11 nm and then increased to 13 nm, the absorbance wavelength was decreased from 528 to 519 nm, and AuNP absorbance at the absorption peak increased from 0.893 to 0.982. The blueshift of the maximum peak of AuNPs demonstrated the successful synthesis of AuNPs. When 0.5 mL trisodium citrate was added, the diameter of AuNPs was larger. This may have been due to the fact that when the concentration of $C_{6}H_{5}O_{7}^{3-}$ was low (0.5 mL) and the concentration of $A_{u}{\mathrm{Cl}}_{4}^{-}$ was high, the surface potential difference of Au^0^ adsorbed little $A_{u}{\mathrm{Cl}}_{4}^{-}$ and the surface potential difference of particles was small, which resulted in AuNPs with a relatively large diameter. When 3 mL trisodium citrate was added, $A_{u}{\mathrm{Cl}}_{4}^{-}$ was reacted exactly to Au^0^. Therefore, AuNPs with the same charge were mutually exclusive based on the electrostatic interaction, resulting in the AuNPs with good dispersion and a diameter of 11 nm. When 4 mL trisodium citrate was added, the agglomeration between the particles was obvious. The reason for this may due to the high concentration of Na^+^, which was easy to neutralize with the negative charge of $C_{6}H_{5}O_{7}^{3-}$ adsorbed on the surface of AuNPs, which made the AuNPs aggregate and the particle size increased slightly.

### 2.3. The Relationship between AuNPs Size and SERS Signal Intensity

The previous studies have demonstrated that aggregated AuNPs could greatly enhance the SERS signal intensity to detect individual molecules. However, the formation of plasmonic near-field “hot spots” were typically achieved in an uncontrollable way with a low spatial density and uneven distribution [28]. In order to conduct a quantitative analysis on the size and density of AuNPs, the relationship between the AuNP size and the SERS signal intensities at 529, 560, 610, 674, 1100 and 1270 cm^−1^ of the CPF molecule were established.

**Figure 3:**
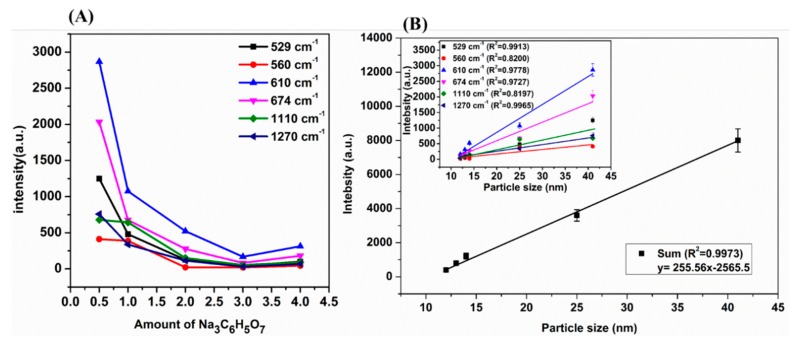
(**A**) The SERS intensity of CPF at 529, 560, 610, 674, 1100 and 1270 cm^−1^ of the CPF molecule. (**B**) The relationship between the SERS characteristic peak intensity of the CPF molecule and the AuNP size.

As shown in Figure 3, with the increase of Na_3_C_6_H_5_O_7_ from 0.5 to 4 mL, the SERS intensity of CPF at 529, 560, 610, 674, 1100 and 1270 cm^−1^ decreased gradually. There were good linear correlations between the SERS characteristic peak intensity at 529, 560, 610, 674, 1100 and 1270 cm^−1^ of the CPF molecule and AuNP diameters with an R^2^ of 0.9913, 0.8200, 0.9778, 0.9727, 0.8197 and 0.9655, respectively. In addition, the value of R^2^ reached 0.9973 between the sum of the SERS characteristic peak intensities and AuNP diameters. The reason might be that when the AuNP diameters were the largest (42 nm in this study), the electric field force among the AuNPs was the strongest and the effects of “hot spots” among the largest AuNPs also reached the strongest, resulting in the strongest SERS signal of CPF [29]. In further study, the AuNPs prepared by 0.5 mL trisodium citrate with a good enhancement effect were selected as the SERS substrates to detect CPF residues in soil.

### 2.4. Quantitative SERS Determination of CPF

**Figure 4:**
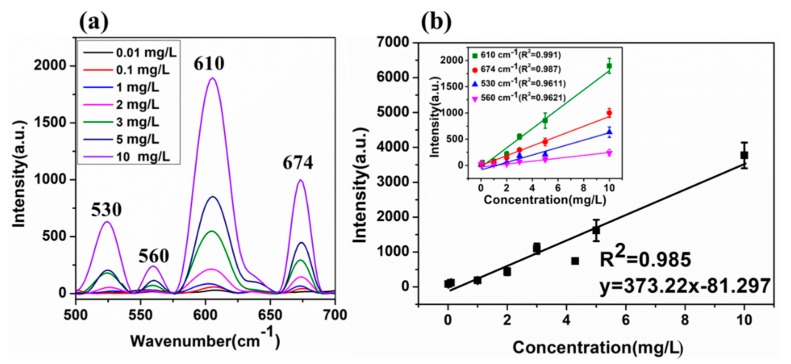
(**a**) The Raman spectra of CPF with AuNPs at different concentrations ranging from 0.01 to 10 mg/L. (**b**) A linear equation of Raman characteristic peak intensity and its concentration at 530, 560, 610, 674 cm^−1^ of the CPF molecule.

To investigate the sensitivity and stability of the prepared AuNPs (42 nm) substrates prepared by 0.5 mL trisodium citrate for the determination of CPF, the representative SERS spectra of CPF solutions at different concentrations ranging from 0.01 to 10 mg/L were obtained (Figure 4a).

Although the Raman intensity largely decreased with dilution of the CPF solution, it was also found that the characteristic peaks at 530, 560, 610, 674 cm^−1^ of the CPF molecules were still identified even when the CPF solution concentration was as low as 10 μg/L. For the quantitative determination of CPF, the linear fit with error bars based on seven spectra was used and the value of R^2^ reached 0.985, which was shown in Figure 4b. It was proved that the synthetic AuNP with ultra-sensitivity and reproducibility was a good SERS substrate for CPF detection. Compared with the results obtained by Qin et al. [23] with the LOD of 0.35 mg/kg, the LOD of CPF was greatly enhanced in this study. 

### 2.5. Quantitative Determination of CPF Residues in Soil

**Figure 5:**
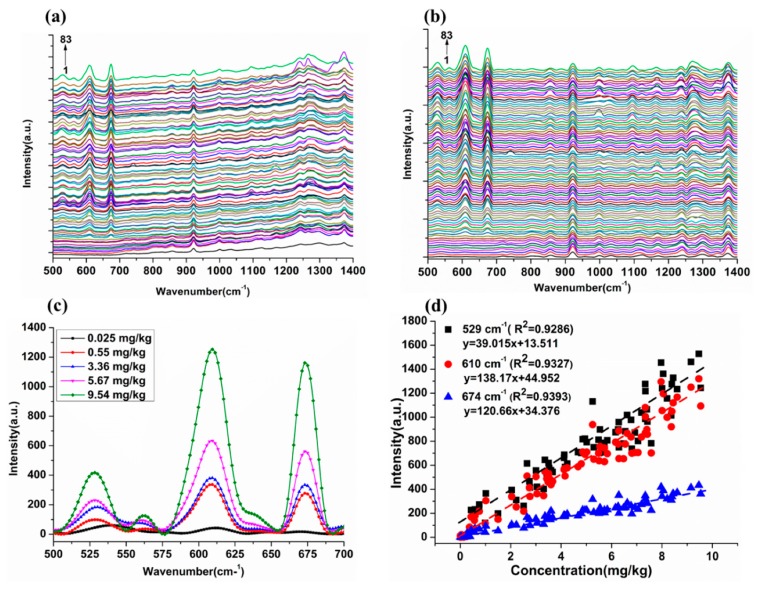
(**a**) The original SERS spectra of 83 samples. (**b**) The SERS spectra after baseline correction (BC) of 83 samples. (**c**) The original SERS spectra after BC of five samples. (**d**) Linear equations of Raman characteristic peak intensities and its concentration at 529, 610 and 674 cm^−1^ of CPF in soil.

The complexity of the soil matrix, such as organic matter, fat and total nitrogen, makes it difficult to detect CPF residues in soil using SERS. In this study, to investigate the feasibility of the AuNP substrate for the detection of CPF in soil, CPF solutions with different concentrations ranging from 0 to 10 mg/L were added to the soil and the CPF residues were extracted from the soil according to QuEChERS method. The representative 500–1400 cm^−1^ SERS spectra of 83 samples are shown in Figure 5a,b and the corresponding concentrations from low to high are given in Table S1.

It can be clearly seen that there was a baseline shift in the original spectra of CPF in soil (Figure 5a). Following the baseline correction (BC), with an increase of CPF concentration in soil from 0.025 to 9.54 mg/kg, the intensity of CPF characteristic peaks at 529, 610 and 674 cm^−1^ increased gradually (Figure 5c), which indicated that the SERS technique could be used for the quantitative determination of CPF in soil, and the characteristic peaks located at 529, 610 and 674 cm^−1^ of CPF molecules were still identified even when the solution concentration was as low as 0.025 mg/kg below the national standard for soil environmental quality (0.05 mg/kg). However, there is still room for the improvement of LOD for reaching the μg/kg level. For the quantitative detection of CPF in soil, the linear fit calibration curves based on 83 spectra were used and the value of R^2^ at 529, 610 and 674 cm^−1^ intensity reached 0.9286, 0.9327 and 0.9393, respectively (Figure 5d). The results proved that CPF residues in soil could be quantitatively determined by the SERS technique. Moreover, it can be seen that the R^2^ of CPF in soil at 529, 610 and 674 cm^−1^ were lower than the CPF in different concentrations. This might due to the influence of soil background matrix.

### 2.6. PLSR Models for Predicting CPF Residues in Soil 

**Figure 6:**
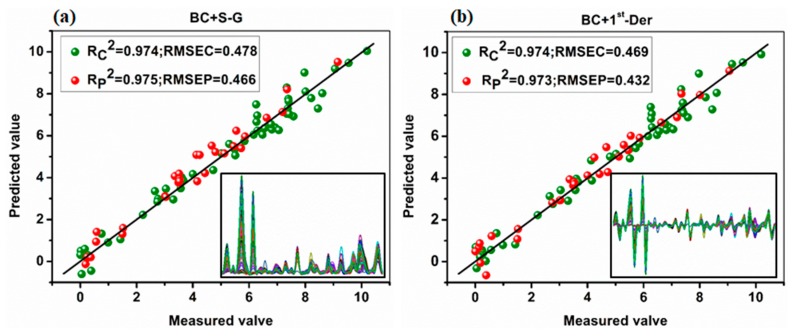
The prediction effect of the PLSR model with different spectral preprocessing methods: (**a**) the SERS spectra processed with BC and S-G treatment; (**b**) the SERS spectra processed with BC and 1st-Der treatment.

In order to improve the detection accuracy, the PLSR prediction model was established based on the full spectra. The SERS spectra of 83 samples were obtained and then pretreated with the BC, Savitzky–Golay smoothing (S-G), 1st-Derivative (1st-Der), multiplicative scatter correction (MSC) and standard normal variation (SNV) respectively, and then modeled by PLSR. The sample set portioning based on the joint x–y distance (SPXY) [30] method was used to separate the soil samples into calibration set and validation set at a ratio of 2:1. The performances of the PLSR models based on full spectra with different pretreatments are shown in Table 4, and the optimum performances of the PLSR models based on full spectra are shown in Figure 6.

Firstly, for CPF concentrations in soil, the PLSR model based on the full spectra performed a better predictive capability than the model established on the Raman intensity of the characteristic peaks at 529, 610 and 674 cm^−1^. The reason for this might be that the PLSR model had the advantages of robustness and flexibility in dealing with a large amount of redundant spectral data. Secondly, from the perspective of the modeling results before BC processing, it can be seen that when the SERS spectra were processed with MSC, the prediction accuracy and stability of the PLSR model was optimum with an R^2^C of 0.947, R^2^P of 0.962 and residual prediction deviation (RPD) of 4.72. A possible explanation is that MSC could eliminate the effect of uneven sample distribution and filling density, which improved the spectral resolution, and reduced the standard deviation between samples for quantitative analysis. However, when the SERS spectra were processed after BC and then processed by SNV, the predictive capability decreased sharply with an R^2^C of 0.947 for calibration set and R^2^P of 0.930 for prediction set. The reason for this could be that the baseline drift with different concentrations of pesticide residues in soil was over-averaged. In general, the PLSR model of the SERS spectra of CPF in soil processed with BC and S-G smoothing achieved the best prediction effect with an R^2^P of 0.977 and an RPD of 4.78.

### 2.7. Model Accuracy Verification 

To verify the accuracy of the determination method of soil pesticide residue performed in this study, CPF (0.6, 4 and 8 mg/L) were added and mixed with soil. Each concentration contained three samples. Second, all the samples were detected by ultra high-performance liquid chromatography (UHPLC) and SERS. Third, the linear regression equations at 674 cm^−1^ and PLS model were used to predict the CPF pesticides in soil, respectively. Table 5 presents the results between the real value and predicted value of CPF pesticides in soil.

According to Table 5, the CPF pesticides in soil could be predicted better using the PLSR model than using the linear regression equations at 674 cm^−1^. The relative standard deviation (RSD) was less than 9.20 and 3.69% for three added concentrations in the two models, respectively. In addition, the recovery % was in the range of 84.0–94.5% and 97.5–103.3%. It is further demonstrated that the application of the SERS technique for the determination of soil pesticide residue is reliable and effective, which shows great potential in the pesticide residue detection of soil.

## 3. Materials and Methods

### 3.1. Chemicals and Instruments

In this experiment, CPF (C_9_H_11_Cl_3_NO_3_PS, 99.5% purity, Chemical Reagent Beijing Co., Ltd., Beijing, China), acetonitrile (C_2_H_3_N), chloroauric acid (HAuCl_4_), trisodium citrate (Na_3_C_6_H_5_O_7_), sodium chloride (NaCl), anhydrous sodium acetate (NaC_2_H_3_O_2_), magnesium sulfate (MgSO_4_·7H_2_O), C_18_ and graphite carbon black (Analytical Purity, Chemical Reagent Beijing Co., Ltd., Beijing, China) were used as chemical reagents. A Raman spectrometer equipped with a 785-nm laser (Opto Trace Technologies, Inc., Suzhou, China) was used to obtain the SERS spectra. Optical absorption measurements of AuNPs were carried out by a TU-1901 Ultraviolet Spectrophotometer (Beijing General Instrument Co., Ltd., Beijing, China). Morphological features of the prepared AuNPs structures were characterized with TEM (Hitachi Ltd., Tokyo, Japan). CPF residues in soil were detected using Agilent 1290 Ultra High-Performance Liquid Chromatography instrument (UHPLC, Agilent Technology Co., Ltd., Santa Clara, CA, USA).

### 3.2. Synthesis of Gold Nanoparticles

In this study, the synthesis of AuNPs at different Na_3_C_6_H_5_O_7_ concentrations was initiated by the rapid addition of 0.5, 1, 2, 3 and 4 mL of 1% Na_3_C_6_H_5_O_7_ into 100 mL boiling HAuCl_4_ at a concentration of 0.01%, respectively. The solution was then heated and stirred continuously for 20 min at the boiling state, respectively. Finally, the prepared gold colloid was placed in a brown jar and stored in dark at room temperature.

### 3.3. Treatment of Soil Samples Containing CPF Residues

The experimental acidic red soil samples were collected from Lishui City, Zhejiang province, China. Firstly, the soil samples were naturally dried and then sieved with a 0.028-mm mesh. Then, different concentrations of CPF ranging from 0 to 10 mg/L were mixed with the soil and air-dried. The method used to extract CPF residues from soil is referred to as QuEChERS [31]. The specific process was as follows. First, 5 mL ultra-pure water was mixed with 10 g soil sample and vortexed for 30 s. Second, 10 mL of 1% acetonitrile was added and vortexed at 400 r/min for 3 min and then 2 min ultrasonic oscillation. Third, the sample was left for 15 min, and then 4 g NaC_2_H_3_O_2_ and 3 g NaCl were added. The mixed solution had a vortex at 400 r/min for 1 min and centrifugal operation at 5000 r/min for 5 min. Fourth, 1.5 mL supernatant, 50 mg *N*-propyl ethylenediamine (PSA), 10 mg graphite carbon black, 150 mg magnesium sulfate and 50 mg C_18_ were added. The supernatant was then centrifuged for 1 min to remove carbohydrates, proteins, fats and other substances. Finally, the solution was centrifuged for 5 min at 5000 r/min and then the supernatant was obtained through a 0.22-μm organic film for SERS measurement and the UHPLC test.

### 3.4. UHPLC Measurement

A UHPLC instrument (Agilent 6410, Agilent Technologies Co., Ltd., Santa Clara, CA, USA) equipped with a column thermostat, an autosampler, a diode array detector and a degasser unit was used to measure CPF samples to validate the SERS method. The analytical column (Agilent ZORBAX SB-C18, 150 mm × 2.1 mm × 3.5 μm) was kept at 30 °C and the elution was operated at 300 nm with a mixture of methanol and water at a ratio of 1:1 and at a flow rate of 0.3 mL/min.

### 3.5. SERS Measurement

All the Raman spectra of CPF in powder and SERS measurements were conducted by an RmTracer-200-HS portable Raman spectrometer system combined with a 785-nm excitation wavelength diode-stabilized stimulator and the acquisition time was 10 s with 3 accumulations.

### 3.6. Modeling Methods

The SERS spectra of CPF samples are greatly interfered with by the fluorescence background. It is critical to remove the fluorescence background from the Raman signals to accurately analyze SERS spectral data. In the present study, in order to investigate the SERS determination of CPF based on AuNPs, each original SERS spectrum was processed by 5 points smoothing filtering using S-G [32] and then BC treatment. For a better quantitative SERS determination of CPF residues in soil in the PLSR model, each original SERS spectrum was processed by 5 points smoothing filtering using S-G and BC, and then pretreated with 1st-Der [33], MSC, SNV [34], respectively.

### 3.7. Spectral Preprocessing Methods

PLSR has been widely applied in data analysis because of its robustness and flexibility in dealing with large amount of redundant spectral data [35]. In PLSR, the spectral matrix was decomposed, the main principal components were acquired, and then each principal component contribution which was identified by the cross-validation root mean square error was calculated. In this study, to establish the PLSR model, the SERS spectral data was X and the CPF concentration tested by UHPLC was Y.

### 3.8. Model Evaluation Index

In the PLSR model, the degree of affinity among variables is expressed by the determinant coefficient (*R*^2^), the degree of accuracy is expressed by the root mean square error (RMSE), and the stability and predictive ability is expressed by the RPD. The closer the *R*^2^ is to 1, the lower the *RMSE*, and the higher the RPD (at least greater than 3) [36], the better the accuracy, stability and predictive ability of the model. In this study, all above-mentioned data analyses were based on OMNIC v8.2 (Thermo, Nicolet, MA, USA), MATLAB R2014a (Natick, MA, USA) and Gaussian v.09 (Gaussian, Inc., Wallingford, CT, USA).

## 4. Conclusions

In this study, we initially described a simple method for preparing ultrasensitive and reproducible AuNPs with different sizes for the quantitative determination of CPF in soil. Furthermore, the relationship between the SERS characteristic peak intensity of the CPF molecule and AuNP diameter in the range of 10–50 nm was investigated. More specifically, the SERS technique could be applied to effectively detect CPF pesticides in soil and the LOD reached 0.025 mg/kg, which is below the national standard for soil environmental quality (0.05 mg/kg). Also, the predictive capability of the PLS model was better than that of the single variable model. It is believed that the prepared AuNP is an excellent substrate for the sensitive SERS determination of pesticide residues in soil, which is favorable for soil remediation and environmental protection. However, there is still room for the practical application of SERS technology in soil pesticide residue detection, for example, the improvement of the SERS sensitivity, and the improvement of LOD for reaching the μg/kg level.

## Figures and Tables

**Table 1:** Surface-enhanced Raman spectroscopy (SERS) investigations for the detection of chlorpyrifos (CPF).

Base	Substrate	Synthetic Procedure	Particle Size	LOD	Ref.
Apple	Au@AgNPs	Na_3_C_6_H_5_O_7_/HAuCl_4_/C_6_H_8_O_6_/AgNO_3_	45 nm	0.14 μg/cm^2^	[19]
Rice	OTR202	No description	50 nm	0.506 mg/L	[20]
Apple	AuNPs	No description	-	0.13 mg/kg	[21]
Peel	AuNPs	HAuCl_4_ (100 mL, 2.5 × 10^−4^ M)/Na_3_C_6_H_5_O_7_ (5 mL, 1%)	25 nm	3.51 ng/cm^2^	[22]
Water	AuNPs	K_2_CO_3_ (1 mL, 0.2 M)/HAuCl_4_ (25 mL, 2.5 × 10^−4^ M)/NH_2_OH·HCl (1 mL, 10.0 mM)	80 nm	10^−6^ M	[23]
Apple	Ag_2_O@AgNPs	SiO_2_ wafers/Ar plasma/physical vapor deposition/PMMA film	80 nm	10^−7^ M	[24]
Apple	AgNPs	AgNO_3_ (4 mL, 1.0 mM)/NaBH_4_ (2.0 mM, 10 mL)	-	0.01 mg/L	[2]
Apple	AgNPs	HOH_3_Cl/NaOH (1.5 × 10^2^ mol/L, 10 mL)/AgNO_3_ (1.11 × 10^3^ mol/L, 90 mL)	-	64 μg/kg	[25]
Apple	AuNPs	No description	20 nm	2.64 mg/cm^2^	[26]

**Table 2:** The vibrational mode of various peaks for CPF.

DFT (cm^−1^)	CPF Powder (cm^−1^)	SERS (cm^−1^)	Assignment
523	530 (w)	529 (s)	υ (P–O)
562	566 (m)	566 (w)	υ (P=S) + υ (C–Cl)
634	630 (vs)	610 (vs)	υ_breathe_ + υ (P=S) + υ (C–Cl)
670	676 (s)	674 (s)	υ_ring_ + δ (C–Cl)
950	969 (m)	964 (vw)	υ (P–O–C)
1119	1100 (s)	1100 (m)	δ (CH_3_)
1243	1237(s)	-	υ_ring_ + υ (C=N)
1283	1277 (s)	1270 (m)	δ (CH_3_)

vs = very strong; s = strong; m = medium; w = weak; υ = stretching; δ = deformable vibration.

**Table 3:** Physical parameters of AuNPs and peak intensities.

Sample	*^a^v*/mL	α *_m_*	*λ_m_*/nm	*r*/nm
a	0.5	0.893	528	42
b	1	0.974	526	25
c	2	0.934	525	14
d	3	0.935	521	11
e	4	0.982	519	13

*^a^v*: the amount of trisodium citrate; *α_m_*: AuNP absorbance at the absorption peak; *λ_m_*: the AuNPs absorption peak wavelength; *r*: AuNP particle size.

**Table 4:** The performances of the partial least squares regression (PLSR) models based on full spectra with different pretreatments.

Baseline	Pretreatment	Calibration		Prediction		
		R^2^C	RMSEC	R^2^P	RMSEP	RPD
Before	S-G ^a^	0.943	0.617	0.954	0.637	4.07
	1st-Der	0.945	0.536	0.960	0.684	3.96
	MSC	0.947	0.684	0.962	0.528	4.72
	SNV	0.947	0.698	0.959	0.495	5.00
After	S-G	0.974	0.437	0.977	0.484	4.78
	1st-Der	0.974	0.469	0.973	0.432	5.81
	MSC	0.960	0.601	0.940	0.502	4.06
	SNV	0.965	0.550	0.930	0.577	3.56

^a^ SG, Savitzky–Golay smoothing; MSC, multiplicative scatter correction; SNV, standard normal variation; 1st-Der, 1st-Derivative; R^2^C and R^2^P, coefficients of determination for calibration and prediction sets, respectively; RMSEC and RMSEP, root mean square errors of calibration and prediction sets, respectively; RPD, residual prediction deviation.

**Table 5:** The precision and accuracy for the determination of CPF pesticides in soil.

Model	Added (mg/L)	UHPLC (mg/L) Mean + SD	Predicted (mg/L) Mean + SD	^a^ RSD (%)	Recovery (%)
674 cm^−1^	0.6	0.562 ± 0.056	0.53 ± 0.032	9.20	88.3
	4	3.52 ± 0.158	3.36 ± 0.231	8.23	84.0
	8	7.63 ± 0.173	7.75 ± 0.229	7.56	94.5
PLS	0.6	0.56 ±0.056	0.58 ± 0.027	3.52	97.5
	4	3.52 ± 0.158	4.13 ± 0.157	3.69	103.3
	8	7.63 ± 0.173	7.83 ± 0.210	2.23	97.8

^a^ SD, standard deviation; RSD, relative standard deviation.

## Supplementary Materials

Supplementary materials can be found at https://www.mdpi.com/1422-0067/20/11/2817/s1.

## References

[1] Hou R.Y., Zhang Z., Pang S., Yang T., Clark J.M., He L. (2016). Alteration of the non-systemic behavior of the pesticide ferbam on tea leaves by engineered gold nanoparticles. Environ. Sci. Technol..

[2] Feng S., Hu Y., Ma L., Lu X. (2017). Development of molecularly imprinted polymers-surface-enhanced Raman spectroscopy/colorimetric dual sensor for determination of chlorpyrifos in apple juice. Sens. Actuat. B Chem..

[3] Ariasestévez M., Lópezperiago E., Martínezcarballo E., Simalgándara J., Mejuto J.C., Garcíarío L. (2008). The mobility and degradation of pesticides in soils and the pollution of groundwater resources. Agric. Ecosyst. Environ..

[4] Kalia A., Gosal S.K. (2011). Effect of pesticide application on soil microorganisms. Arch. Agron. Soil Sci..

[5] Camenzuli L., Scheringer M., Hungerbã¼Hler K. (2016). Local organochlorine pesticide concentrations in soil put into a global perspective. Environ. Poll..

[6] Rejczak T., Tuzimski T. (2015). Recent Trends in Sample Preparation and Liquid Chromatography/Mass Spectrometry for Pesticide Residue Analysis in Food and Related Matrixes. J. AOAC Int..

[7] Gonçalves C., Alpendurada M.F. (2004). Solid-phase micro-extraction-gas chromatography-(tandem) mass spectrometry as a tool for pesticide residue analysis in water samples at high sensitivity and selectivity with confirmation capabilities. J. Chromatogr. A.

[8] Liu M., Hashi Y., Song Y., Lin J.-M. (2005). Simultaneous Determination of Carbamate and Organophosphorous Pesticides in Fruits and Vegetables by Liquid Chromatography-mass Spectrometry. J. Chromatogr. A.

[9] Önal A. (2006). A review: Current analytical methods for the determination of biogenic amines in foods. Food Chem..

[10] Didier B., Thierry W., Magalie L.J., Rapha L.A., Luc R., Barthès B.G. (2009). Determination of soil content in chlordecone (organochlorine pesticide) using near infrared reflectance spectroscopy (NIRS). Environ. Poll..

[11] Luo Q., Lai J., Ping Q., Wang X. (2018). An ultrasensitive fluorescent sensor for organophosphorus pesticides detection based on RB-Ag/Au bimetallic nanoparticles. Sens. Actuat. B Chem..

[12] Nie S., Emory S.R. (1997). Probing Single Molecules and Single Nanoparticles by Surface-Enhanced Raman Scattering. Science.

[13] Yang J., Ryckman J.D., Ciesielski P.N., Escobar C.A., Jennings G.K., Weiss S.M. (2011). Patterned nanoporous gold as an effective SERS template. Nanotechnology.

[14] Fu Y., Kuppe C., Valev V.K., Fu H., Zhang L., Chen J. (2017). Surface Enhanced Raman Spectroscopy: A Facile and Rapid Method for the Chemical Components Study of Individual Atmospheric Aerosol. Environ. Sci. Technol..

[15] Sharma B., Frontiera R.R., Henry A.I., Ringe E., Duyne R.P.V. (2012). SERS: Materials, applications, and the future. Mater. Today.

[16] Huiyuan G., Zhiyun Z., Baoshan X., Arnab M., Craig M., White J.C., Lili H. (2015). Analysis of Silver Nanoparticles in Antimicrobial Products Using Surface-Enhanced Raman Spectroscopy (SERS). Environ. Sci. Technol..

[17] Alvarez-Puebla R.A., Liz-Marzan L.M. (2010). Environmental applications of plasmon assisted Raman scattering. Energy Environ. Sci..

[18] Xu S., Man B., Jiang S., Wang J., Wei J., Xu S., Liu H., Gao S., Liu H., Li Z. (2015). Graphene/Cu nanoparticle hybrids fabricated by chemical vapor deposition as surface-enhanced Raman scattering substrate for label-free detection of adenosine. ACS Appl. Mater. Interfaces.

[19] Bianhua L., Guangmei H., Zhongping Z., Renyong L., Changlong J., Suhua W., Ming-Yong H. (2012). Shell thickness-dependent Raman enhancement for rapid identification and detection of pesticide residues at fruit peels. Anal. Chem..

[20] Huang S., Hu J., Ping G., Liu M., Wu R. (2015). Rapid detection of Chorpyriphos Residues in rice by Surface-Enhanced Raman Scattering. Anal. Methods.

[21] Zhai C., Li Y., Peng Y., Xu T. (2015). Detection of chlorpyrifos in apples using gold nanoparticles based on surface enhanced Raman spectroscopy. Int. J. Agric. Biol. Eng..

[22] Chen J., Huang Y., Kannan P., Zhang L., Lin Z., Zhang J., Chen T., Guo L. (2016). Flexible and Adhesive Surface Enhance Raman Scattering Active Tape for Rapid Detection of Pesticide Residues in Fruits and Vegetables. Anal. Chem..

[23] Xu Q., Guo X., Xu L., Ying Y., Wu Y., Wen Y., Yang H. (2017). Template-Free Synthesis of SERS-Active Gold Nanopopcorn for Rapid Detection of Chlorpyrifos Residues. Sens. Actuat. B Chem..

[24] Li C., Cheng Y., Xu S., Chao Z., Zhen L., Liu X., Jiang S., Huo Y., Liu A., Man B. (2017). Ag_2_O@Ag core-shell structure on PMMA as low-cost and ultra-sensitive flexible surface-enhanced Raman scattering substrate. J. Alloy. Compd..

[25] Chen Z., Peng Y., Li Y., Chao K. (2017). Extraction and identification of mixed pesticides’ Raman signal and establishment of their prediction models. J. Raman Spectros..

[26] Chen J., Dong D., Ye S. (2018). Detection of pesticide residue distribution on fruit surfaces using surface-enhanced Raman spectroscopy imaging. RSC Adv..

[27] Zhu W.L., Tan X.J., Puah C.M., Gu J.D., Jiang H.L., Chen K.X., Felder C.E., Silman I., Sussman J.L. (2000). How Does Ammonium Interact with Aromatic Groups? A Density Functional Theory (DFT/B3LYP) Investigation. J. Phys. Chem. A.

[28] Zhi Y.B., Xin L., Chen Y., Wu Y., Chan H.L.W., Dai J., Dang Y.L. (2014). Quantitative SERS detection of low-concentration aromatic polychlorinated biphenyl-77 and 2,4,6-trinitrotoluene. J. Hazardous Mater..

[29] Li J.F., Zhang Y.J., Ding S.Y., Panneerselvam R., Tian Z.Q. (2017). Core–Shell Nanoparticle-Enhanced Raman Spectroscopy. Chem. Rev..

[30] Li M., Wang J., Du F., Diallo B., Xie G.H. (2017). High-throughput analysis of chemical components and theoretical ethanol yield of dedicated bioenergy sorghum using dual-optimized partial least squares calibration models. Biotechnol. Biofuels.

[31] Łozowicka B., Rutkowska E., Jankowska M. (2017). Influence of QuEChERS modifications on recovery and matrix effect during the multi-residue pesticide analysis in soil by GC/MS/MS and GC/ECD/NPD. Environ. Sci. Poll. Res..

[32] Bromba M.U.A., Ziegler H. (1981). Application Hints for Savitzky-Golay Digital Smoothing Filters. Anal. Chem..

[33] Fernandez D.C.D.R., Boom P.D., Zingg D.W. (2017). Corner-corrected diagonal-norm summation-by-parts operators for the first derivative with increased order of accuracy. J. Comput. Phys..

[34] Fearn T., Riccioli C., Garrido-Varo A., Guerrero-Ginel J.E. (2009). On the geometry of SNV and MSC. Chemomet. Intell. Lab. Syst..

[35] Zhang C., Jiang H., Liu F., He Y. (2017). Application of Near-Infrared Hyperspectral Imaging with Variable Selection Methods to Determine and Visualize Caffeine Content of Coffee Beans. Food Bioprocess Technol..

[36] D’Acqui L.P., Pucci A., Janik L.J. (2010). Soil properties prediction of western Mediterranean islands with similar climatic environments by means of mid-infrared diffuse reflectance spectroscopy. Eur. J. Soil Sci..

