# Supplementary material for: Gold Nanoparticles with Different Particle Sizes for the Quantitative Determination of Chlorpyrifos Residues in Soil by SERS
Source: Int J Mol Sci. 2019 Jun 10;20(11):2817. doi: 10.3390/ijms20112817 (PMC6600568; doi:10.3390/ijms20112817)
Supplement: Supplementary file 1 [file ijms-20-02817-s001.pdf]

Table S1. The corresponding concentrations form low to high of 83 samples.

Number	Concentration (mg/kg)	Number	Concentration (mg/kg)	Number	Concentration (mg/kg)
1	0.025	29	3.361	57	6.290
2	0.056	30	3.430	58	6.296
3	0.132	31	3.488	59	6.478
4	0.169	32	3.510	60	6.550
5	0.185	33	3.522	61	6.626
6	0.157	34	3.529	62	6.777
7	0.048	35	3.570	63	6.807
8	0.185	36	3.600	64	6.931
9	0.241	37	3.666	65	7.056
10	0.277	38	4.119	66	7.192
11	0.369	39	4.128	67	7.340
12	0.386	40	4.156	68	7.346
13	0.424	41	4.243	69	7.348
14	0.559	42	4.673	70	7.402
15	0.582	43	4.803	71	7.423
16	0.756	44	4.858	72	7.578
17	0.986	45	4.949	73	7.970
18	1.013	46	5.017	74	8.008
19	1.499	47	5.251	75	8.055
20	2.039	48	5.294	76	8.210
21	2.224	49	5.488	77	8.374
22	2.519	50	5.509	78	8.397
23	2.651	51	5.545	79	8.447
24	2.742	52	5.717	80	8.605
25	2.765	53	5.724	81	9.153
26	3.001	54	5.835	82	9.465
27	3.043	55	6.160	83	9.540
28	3.306	56	6.265		
